# Leaf photosynthetic function duration during yield formation of large-spike wheat in rainfed cropping systems

**DOI:** 10.7717/peerj.5532

**Published:** 2018-09-28

**Authors:** Lifang Wang, Jutao Sun, Chenyang Wang, Zhouping Shangguan

**Affiliations:** 1 Agronomy College/National Engineering Research Center for Wheat, Henan Agricultural University, Zhengzhou, China; 2 College of Tobacco Science, Henan Agricultural University, Zhengzhou, China; 3 State Key Laboratory of Soil Erosion and Dryland Farming on the Loess Plateau, Northwest A&F University, Yangling, China

**Keywords:** Large-spike wheat, Grain yield, Drought resistance, Photosynthesis characteristics

## Abstract

Improving photosynthetic capacity significantly affects the yield of wheat (*Triticum aestivum* L.) in rainfed regions. In this study, the physiological characteristics of eight large-spike wheat lines were compared with a multiple-spike cultivar as a control (CK) in a field over two consecutive seasons: 2010–2012. The tillering peak was 7–21 d after returning green for line 2040, the average rate of decline of relative water content was slower, and the average duration time of photosynthetic rate was longer than CK *in vitro*. There was a strong linear and positive correlation between photosynthetic rate and root activity at jointing, flowering, and grain-filling stages. In addition, average yields were higher in large-spike lines than CK (multiple-spike cultivar). The results suggest that large-spike lines might have greater water retaining capacity during yield formation under rainfed conditions.

## Introduction

Wheat (*Triticum aestivum* L.) is the most important staple winter cereal crop in the North China Plain and water shortage is a serious issue threatening the sustainable development of agriculture in this area (Zheng et al., 2014). Drought tolerance is considered a valid breeding target in the stabilization of crop performance by breeders (Riccardi et al., 2016). With the world population continuously increasing, much of the future food will have to come from rainfed areas (Turral, Svendsen & Faures, 2010). The imperative to develop drought-resistant crops is intensifying due to increasingly limited water supplies for crop production.

Leaf senescence comprises a series of biochemical and physiological events from the fully expanded state until death. The green leaf area duration is one of the important physiological traits with implication for yield potential related to increasing assimilate (i.e., source) availability. The leaf duration after full expansion depends strongly on the water conditions and crop species; some researchers have reported that the post-anthesis senescence in cereals affects the whole plant, with organs closest to the developing grains (i.e., flag leaves and glumes) generally senescing last (Distelfeld, Avni & Fischer, 2014). The leaf relative water content (RWC) was found to be correlated to the drought resistance and water saving of wheat cultivars (Dong et al., 2008; Khakwani et al., 2012). Using RWC and leaf senescence as indirect selection criteria for wheat grain yield is an interesting alternative approach because these traits are easily and rapidly screened, and relatively inexpensive, and we can gain a partial understanding of many physiological mechanisms that confer drought tolerance and lead to the development of wheat better adapted to environments.

Photosynthesis plays an important role in modern winter wheat cultivars, especially in under rainfed conditions, and it is the most crucial source of biomass accumulation in plants; the chlorophyll content of leaves is one of the major indicators of photosynthetic capability of plant tissues (Pietrini et al., 2017; Nageswara Rao, Talwar & Wright, 2001). Large-spike wheat is characterized by large spikes, high numbers of grains per plant, and high yield potentials (Wang, Chen & Shangguan, 2016), and it also shows greater physiological advantages in root activity (measured by triphenyl tetrazolium chloride (TTC) method), photosynthetic properties, and the number of secondary roots compared with multiple-spike cultivars (Guo et al., 2009; Wang et al., 2010). The photosynthetic function duration of leaves is closely correlated to grain yield of wheat, and previous studies have focused on the yield and photosynthetic traits at growth period (Raven & Griffiths, 2015; Gaju et al., 2016; Merchuk-Ovnat et al., 2016). Few integrated studies on the variation of photosynthetic function duration following the time *in vitro* and the formation of biomass and yield in rainfed environments are available, and poorly investigated in large-spike wheat.

The main objectives of this study were to: (1) investigate the differences in the dynamic changes of chlorophyll relative value (SPAD), RWC, and photosynthetic rate (*P*_n_) parameters following time *in vivo* between eight large-spike lines and one control cultivar, (2) analyze the relationship between TTC and *P*_n_ of large-spike lines and (3) evaluate the changes in photosynthetic pigments, single spike weight, and leaf + stem + sheath weight between the same eight large-spike lines and the control cultivar. The findings should provide some theoretical foundations to guide breeders in selecting high drought-resistant wheat materials, and to improve production potential of large-spike wheat under rainfed conditions.

## Materials and Methods

### Plant materials

In our study, eight new high-yield and large-spike wheat lines (2005, 2013, 2026, 2036, 2037, 2038, 2039 and 2040) were used, which had been tested in the Shaanxi provincial wheat variety trial test in 2009 and the Shaanxi provincial wheat variety regional test in 2010, and these lines were bred through many generations over many years. The winter wheat cultivar of Xi’nong 979 (*Triticum aestivum* cv. Xi’nong 979) has been planted across large areas of the Huang-Huai-Hai production region of winter wheat (Wang & Shangguan, 2015), the detailed characteristics of these materials were provided in Table 1 and would be analyzed in the part of discussion. The seeds were also sown in Sufang Town, Wugong City, Shaanxi Province, northwestern China (Wang, Chen & Shangguan, 2016).

**Table 1:** Main characteristics of wheat materials used in present study.

Materials code	Number of spikes (×10^4^ ha^−1^)	Number of grains per spike	Kernel weight per spike (g)	Yield (kg ha^−1^)	Other characters
CK	654–763	30–51	1.1–2.3	8,659.3–10,598.9	Compact plant type
2005	371–428	45–83	1.5–4.2	7,530.4–9,118.2	–
2013	339–406	39–93	1.3–5.1	6,272.6–10,434.2	Stable types of head progeny row nursery
2026	404–493	31–70	1.5–3.5	7,795.6–9,870.1	–
2036	376–484	30–58	1.6–3.0	9,238.1–10,918.1	Lodging-resistance, shorter awn
2037	455–511	38–68	1.3–3.6	10,830.8–12,714.4	Good disease-resistance
2038	331–642	45–74	1.7–3.5	7,700.6–14,778.2	Good light transmittance, shorter awn
2039	412–541	45–82	1.9–4.0	9,093.4–11,677.8	–
2040	446–629	30–62	1.5–3.4	8,960.4–12,954.7	Leaves and stems are green when the grain is mature

### Culture conditions and experiment design

The field experiment was conducted in the Institute of Soil and Water Conservation, Chinese Academy of Sciences and Ministry of Water Resources, Yangling, Shaanxi (34°16′N, 108°04′E) during the winter wheat growing seasons (October–June) in 2010–2011 and 2011–2012. The experiment was located in the sub-humid warm temperate continental monsoon climate zone and generally had flat topography. The soil of the experiment was a well aerated Eum-orthic Anthrosol soil characterized by favorable permeability, strong water and nutrient-retaining capacities, and wide crop adaptation. There were similar precipitation (231.1 and 229.8 mm) trends and other climate conditions during the two wheat growing seasons, so we chose to analyze the data from 2011–2012.

The field experiment was a randomized block design involving nine treatments, and each plot size was 2 m × 2 m with 10 rows (20 cm spacing) of wheat sown at 110 seeds/row. Surrounding the experimental field, there were five guard rows of wheat. Plants were sampled from the central rows in each plot. Each year, winter wheat was planted on 10 October and the same quantity of chemical fertilizer (360 kg hm^−2^ N and 70 kg hm^−2^P_2_O_5_) was applied to the top 20 cm of soil before planting. During the growing period, no irrigation and fertilizer were applied; the site was plowed to bury weeds and pests before sowing, and weeds were hand-hoed several times during the growing period.

### Population tiller measurement

In each plot, six rows of wheat were chosen and tagged at sowing stage, and the number of tillers were counted at seedling and tillering stages before winter. After wintering, wheat tiller number data were collected at intervals of 7 d from returning green stage to heading stage. The last survey date of the number of spikes was at mature stage.

**Figure 1:**
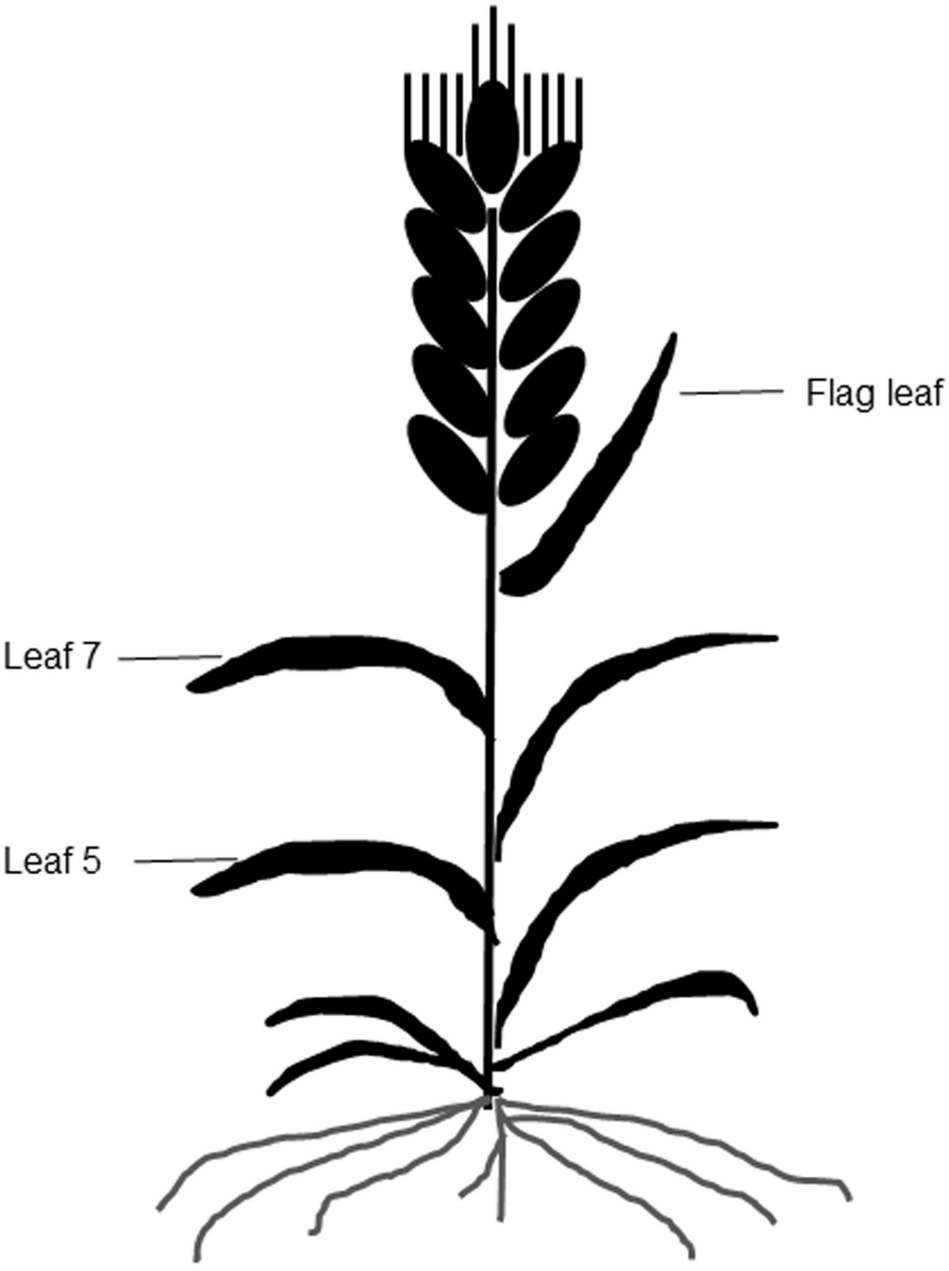
A diagram of different leaf positions in this study.

### Relative value of chlorophyll measurements for intact expanded and detached leaves

A portable chlorophyll meter (SPAD 502; Minolta Camera Co., Osaka, Japan) was used to take SPAD values from the three uppermost fully expanded leaves of leaf 5, leaf 7, and the flag leaf on each plant at 10-d intervals (Fig. 1). A total of six plants were measured per plot, and three SPAD values per leaf, including one value around the midpoint of the leaf blade and two values at 3 cm away from the midpoint were averaged as the mean SPAD value of the leaf (Peng et al., 1993).

From a total of six repeat plants, leaf 5, leaf 7, and the flag leaf were cut when fully expanded and immediately placed in Petri dishes containing distilled water, and cultured in the plant ZPW-280B (DongTuo, Inc., Heilongjiang, China) incubator *in vitro*. During growth, the photoperiod was 12 h (day, 100−120 µmol m^−2^ s^−1^)/12 h (night), temperature was 25 ± 2/18 ± 2 °C (day/night), and relative humidity was 60%–70% (day/night). Measurement methods and measured parts of detached leaves were the same as for intact expanded leaves.

### Photosynthetic rate measurement

For *in vitro P*_n_ measurement, the leaves were separated from plants and preserved in liquid nitrogen immediately, the interval times of measure were 30 min (jointing and flowering stages) and 10 min (grain-filling stage) during 09:00−11:00 AM on clear days with a wind speed below 1 m s^−1^.

The *P*_n_ of fully expanded top-down penultimate and flag leaves for all lines and cultivars was measured with a LI-6400XT Portable Photosynthesis System (LI-6400, *Li-Cor*, Inc., Lincoln, Nebraska, USA) equipped with 2 cm × 3 cm leaf chamber and integrated light source. For the measurement, leaves were intact with no sign of insect or disease attack and the upper one-third of leaves was used. While being measured, the settings were leaf chamber temperature of 25 °C, mean CO_2_ concentration of 382.6 ±  2.5 µL L^−1^, PAR generated by the LED (mixed red and blue) system of 1,300 µmol m^−2^ s^−1^, humidity of 53%–56%, and the gas flow rate of 5 mL min^−1^. The *P*_n_ was measured for 3 min until *P*_n_ and transpiration rates stabilized; for each plot, three randomly selected and fully expanded leaves were measured.

### Relative water content measurement

The RWC was measured following the methodology of Barr & Weatherley (1962). First, six disc samples were cut from the youngest fully expanded leaf at jointing, flowering, and grain-filling stages, and immediately weighed to obtain their fresh weight (FW). Second, the same discs were placed on distilled water for 24 h in Petri dishes in darkness and, after gentle blotting to remove excess water, they were weighed to obtain turgid weight (TW). Finally, discs were weighed after drying at 60 °C for 48 h until constant dry weight (DW) was reached. Using these three parameters, RWC was calculated using the following Eq. (1):

(1) \begin{eqnarray*}& \mathrm{RWC}(\text{%})=(\mathrm{FW}-\mathrm{DW})/(\mathrm{TW}-\mathrm{DW})\times 100.\end{eqnarray*}

The RWC was measured at 30-min intervals at jointing and flowering stages, and 10-min intervals at grain-filling stage.

### Root activity measurement

In the field, the root system was excavated to a size of 20 cm (length) × 20 cm (width) × 30 cm (depth) at the sampling periods of jointing, flowering, and grain-filling stages. The wheat roots were washed with deionized water and excised at five cm from the root tips. Then, root activity was determined by the TTC method (Lindström & Nyström, 1987).

### Photosynthetic pigment measurements

Immediately after the leaf had fully expanded, six leaves were collected from each plot, and each leaf was soaked with 80% acetone and the concentrations of carotenoids (Car), chlorophyll (Chl) *a*, and Chl *b* were determined by measuring absorbance at 663, 646, and 470 nm, respectively, using an UV-2300 spectrophotometer (Techcomp, Inc., Shanghai, China). The Chl concentrations and contents were calculated using the following formulae (Arnon, 1949):

(2) \begin{eqnarray*}& {\mathrm{C}}_{\mathrm{a}}=(12.21\times {\mathrm{A}}_{663})-(2.81\times {\mathrm{A}}_{646})\end{eqnarray*}

(3) \begin{eqnarray*}& {\mathrm{C}}_{\mathrm{b}}=(20.13\times {\mathrm{A}}_{646})-(5.03\times {\mathrm{A}}_{663})\end{eqnarray*}

(4) \begin{eqnarray*}& {\mathrm{C}}_{\mathrm{x}\bullet \mathrm{c}}=(1000\times {\mathrm{A}}_{470}-3.27\times {\mathrm{C}}_{\mathrm{a}}-104\times {\mathrm{C}}_{\mathrm{b}})/229\end{eqnarray*}

(5) \begin{eqnarray*}& \mathrm{Chl~ contents}~({\mathrm{mg~ g}}^{-1})=(\mathrm{C}\times {\mathrm{V }}_{\mathrm{ T}}\times n)/\mathrm{FW}\times 1000\end{eqnarray*}

where C_a_, C_b_, and C_x•c_ are the concentrations of Chl *a*, Chl *b*, and Car, respectively; C is the Chl concentration (mg L^−1^); V_T_ is the volume of extracting solution (mL); FW is the fresh weight of the leaves, and *n* is the dilution multiple.

### Dry weight measurement

In each plot, 100 plants that had headed and flowered on the same days were chosen and tagged, and flowering date was defined as the time when 50% of the plants had flowered (Wang & Shangguan, 2015). Ten plants were collected as samples approximately every 5 d from the first day after the onset of flowering. Every plant was divided into single spike and leaf + stem + sheath parts. Then the two sample parts were used for DW measurement immediately after sampling by deactivating at 105 °C and then drying at 80 °C to a constant weight.

### Yield trait measures

At maturity, 20 plants were randomly selected in each plot for measurement of spike length, number of grains per spike, kernel weight per spike, number of spikes, and 1,000-grain weight. At the same time, six rows (one m length) were selected for estimation of grain yield, excluding borders in each plot.

### Statistical analysis

The data in the tables are the average value of three replicates in the form of means ± SE (standard error). The significant differences (*P* < 0.05) were tested by SPSS ver. 14.0 (SPSS Inc., Chicago, IL, USA), and the differences among the treatments were tested by Duncan’s multiple range test.

## Results

### Dynamic changes in tiller death rate

**Figure 2:**
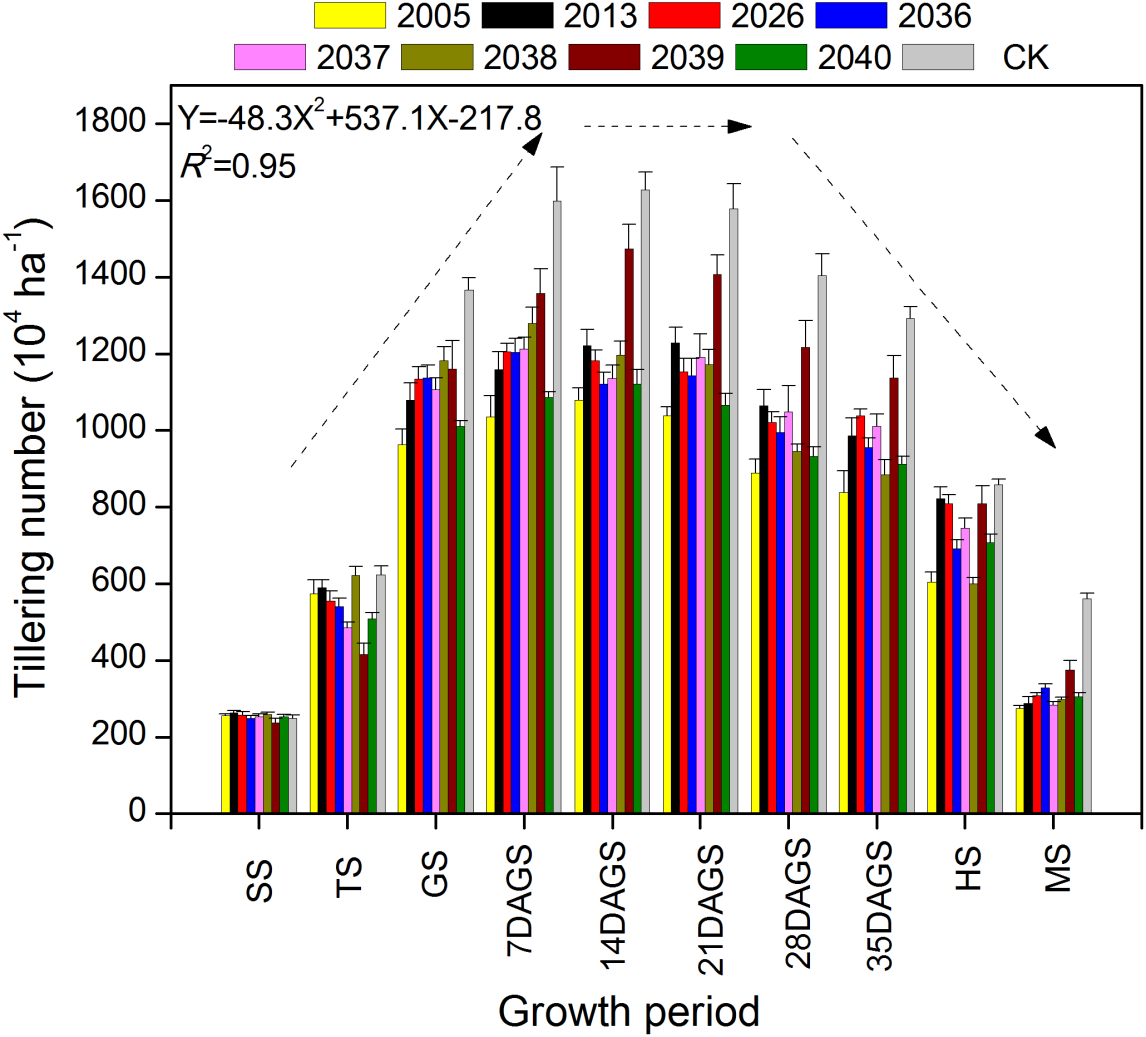
The dynamic changes in tiller number in eight large-spike wheat lines and one multiple- spike cultivar (Xi’nong 979) from seeding to mature stage. SS, seeding stage; TS, tillering stage before winter; GS, returning green stage; 7DAGS, 14DAGS. 21DAGS, 28DAGS, and 35DAGS indicate 7, 14, 21, 28, and 35 d after returning green stage, respectively; HS, heading stage; MS, mature stage.

During the tillering period of seeding–heading and mature stage, there were fewer tillers for large-spike lines than for Xi’nong 979 (Fig. 2). There were different tillering peaks for different wheat materials, the peak for lines 2005, 2039, 2040 and CK was 2 weeks after returning green, and that for line 2013 was 3 weeks after returning green. Although there were two tillering peaks during the growth periods for lines 2036 and 2037, they were 7 d and 3 weeks after returning green. At mature stage, there was twice the number of tillers for Xi’nong 979 compared with large-spike lines, showing their obvious differences in tiller death rates.

### Time courses of declination of chlorophyll relative values

**Figure 3:**
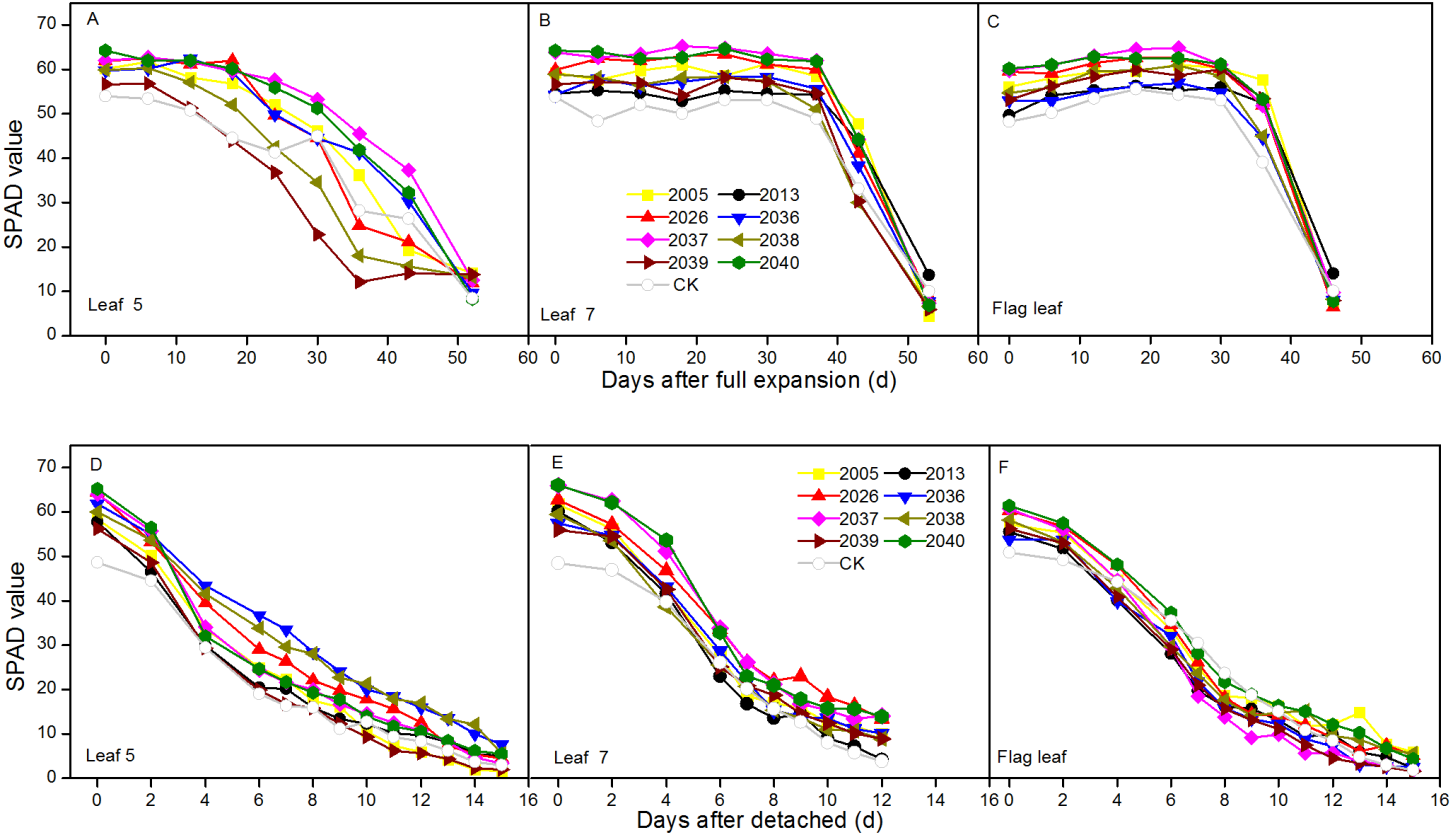
Time courses of chlorophyll relative value (SPAD) decline in fully expanded (A–C) and detached (D–F) leaves at different positions (leaf 5, leaf 7, and flag leaf) for different wheat materials.

The dynamic changes of SPAD were measured in intact expanded and detached leaves at different leaf positions for wheat lines. For the *in vivo* situation, the SPAD peaks were at different positions for wheat lines, the peaks for leaf 5 were around 0 and 6 d after expansion, and the highest values of leaf 7 and the flag leaf were around 24 d. In intact expanded leaves, the degenerative processes of SPAD values for leaf 7 and flag leaf included a relative steady phase and a rapid declining phase, and these were observed in the different wheat lines. The SPAD of leaf 5 showed a uniform decline for the different lines; the average SPAD of leaf 5, leaf 7, and the flag leaf of large-spike lines was 4.29, 6.53, and 6.04 higher than CK, respectively (Figs. 3A–3C).

In the situation *in vitro*, SPAD values showed continuous declines, and the average values of leaf 7 and flag leaf of large-spike lines were 5.64 and 4.62 higher than CK, respectively (Figs. 3D–3F).

### Time courses of RWC and *P*_n_ decline

**Figure 4:**
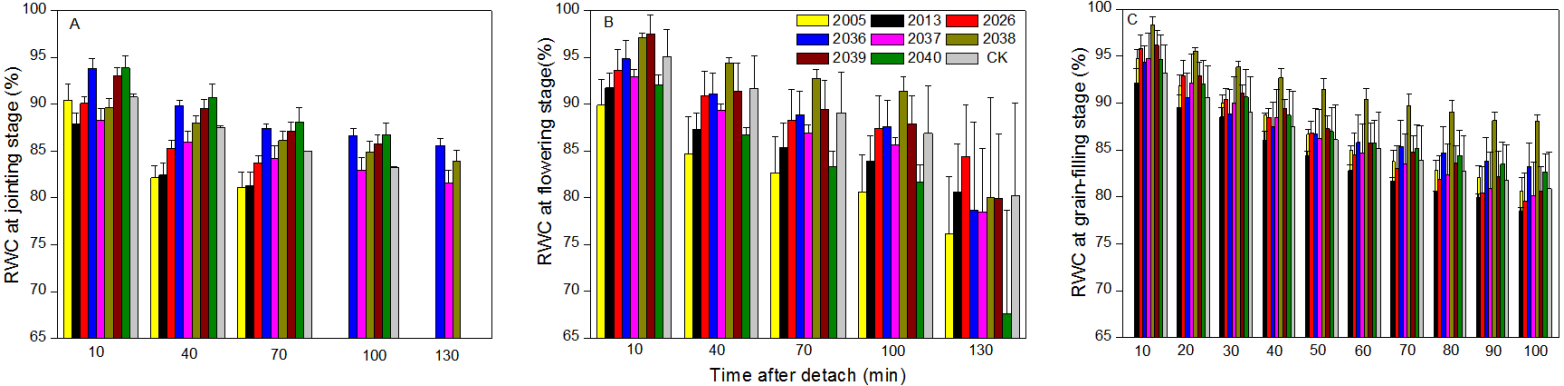
Time courses of relative water content (RWC) decline in detached leaves (A–C) at jointing, flowering, and grain-filling stages for different wheat materials.

**Figure 5:**
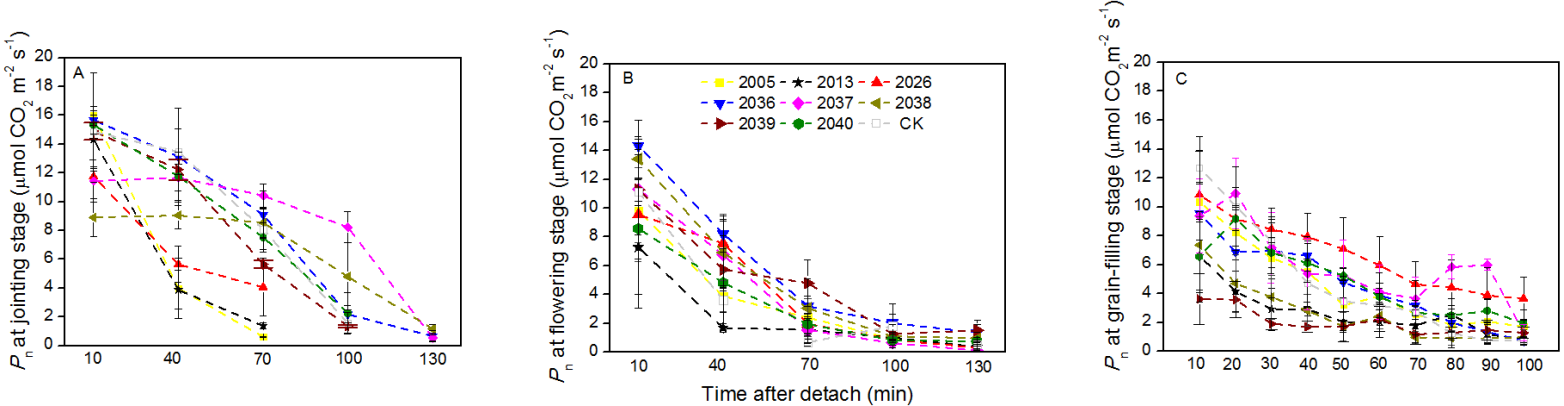
Time courses of photosynthetic rate (*P*_n_) decline in detached leaves (A–C) at jointing, flowering, and grain-filling stages for different wheat materials.

At the jointing stage, the leaf RWC of large-spike lines and CK showed declining trends; the rate of decline of leaf RWC for lines 2037, 2038 and 2040 was 1.74%, 2.32%, and 0.10% lower than for CK after 70 min *in vitro* (Fig. 4A). The *P*_n_ of lines 2036, 2037, and 2038 was higher than for CK, and values were 0.65, 0.55 and 1.18 µmol CO_2_ m^−2^s^−1^ after 130 min *in vitro*, respectively, compared to a negative *P*_n_ of CK for the same times. The average duration times of *P*_n_ of lines 2037, 2038, and 2040 were longer than for the other lines and CK (Fig. 5A), indicating that the three lines had strong drought resistance.

At the flowering stage, the leaf RWC in all wheat lines slowly declined, and the values for lines 2013 and 2026 were higher than CK after 130 min *in vitro*; the decrease amplitudes of leaf RWC for lines 2005, 2013, 2026, and 2037 were smaller than for CK after 10–130 min *in vitro*, showing that they maintained a good water balance (Fig.  4B). The declining trend of *P*_n_ in eight lines and CK was stronger at flowering than at jointing stage; the *P*_n_ of lines 2036, 2038, 2039, and 2040 were higher than CK after 130 min *in vitro*; the decrease in amplitudes of *P*_n_ in lines 2005, 2013, 2026, 2039, and 2040 were smaller than CK after 10–130 min *in vitro* (Fig. 5B), showing that they had a long photosynthetic duration.

At the grain-filling stage, the leaf RWC of all lines was within 78–90%; the values of lines 2036, 2038, and 2040 were higher than CK after 100 min *in vitro*, and decreases in amplitudes of the RWC of these lines were smaller than CK after 10–100 min *in vitro* (Fig. 4C). The variation tendencies of *P*_n_ for wheat materials differed, except for line 2036, the values of the large-spike lines were higher than for CK after 100 min *in vitro*, and the decrease amplitudes of *P*_n_ in all large-spike lines were lower than CK after 10–100 min *in vitro* (Fig. 5C), showing that large-spike lines had strong drought resistance and good water retaining capacity.

### Relationship between *P*_n_ and TTC

**Figure 6:**
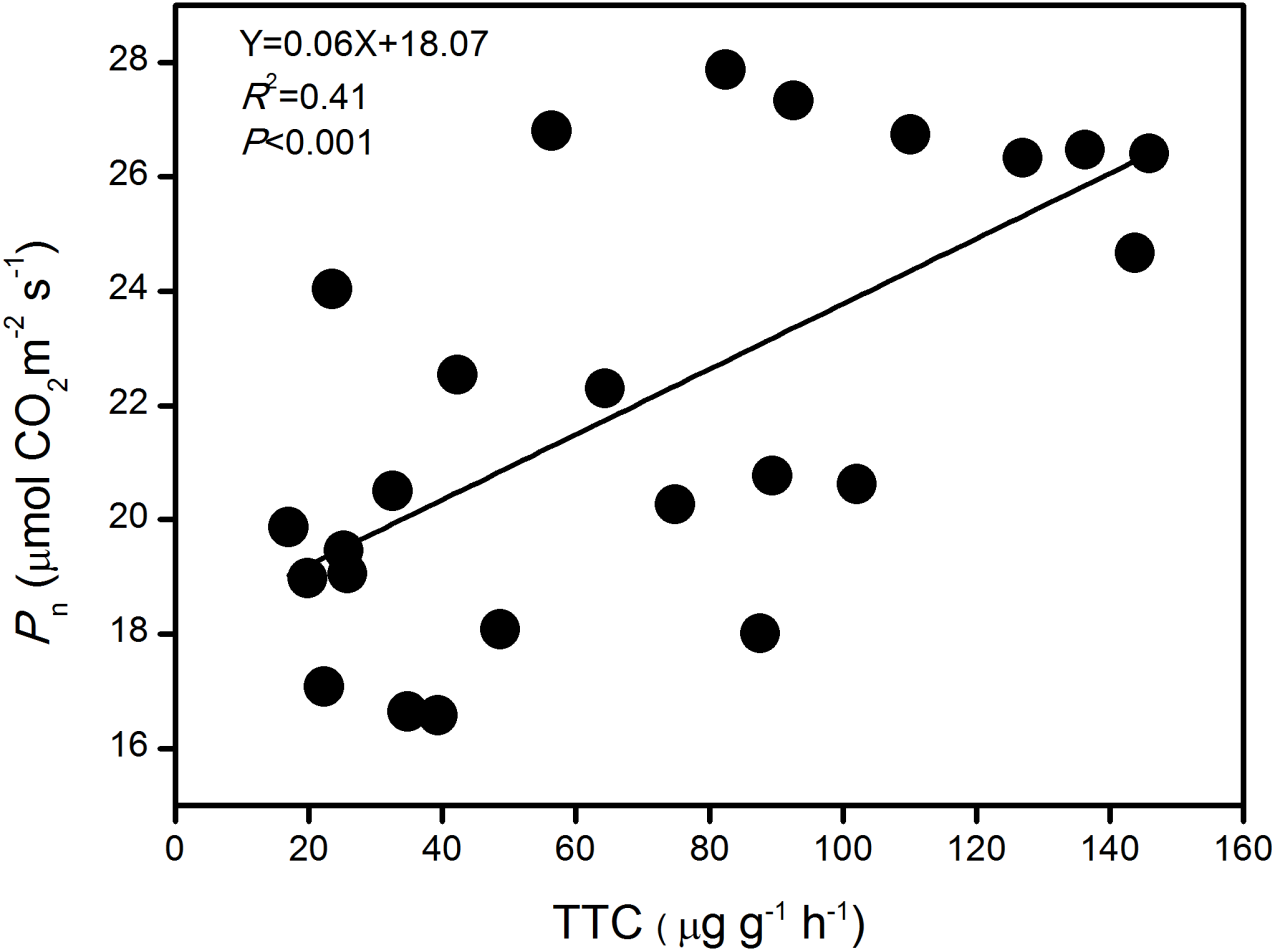
The relationship between photosynthetic rate (Pn) and root activity (TTC) of large-spike wheat lines during the growth period.

The pooled analysis showed a strong linear and positive correlation between *P*_n_ and TTC of large-spike lines at jointing, flowering, and grain-filling stages in this rainfed environment (*P* < 0.001; Fig. 6). The results indicate that TTC might be a good tool for indirect assessment of *P*_n_.

### Photosynthetic pigment contents

The differences in Chl *a* content between large-spike lines and CK were not significant (Table 2). At the jointing stage, the Chl *a* contents of lines 2036, 2037, 2039, and 2040 were higher than CK; the Chl *b* contents of large-spike lines were all lower than CK; and the Car contents of lines 2026, 2036, 2037, 2039, and 2040 were higher than CK. At heading stage, the Chl *a* and *b* contents of lines 2026, 2036, 2037, 2039, and 2040 were higher than CK; and the Car contents of lines 2026, 2036, 2037, and 2040 were higher than CK. At the flowering stage, the Chl *a*, Chl *b*, and Car contents of lines 2026 and 2040 were higher than CK. At the grain-filling stage, the Chl *a*, Chl *b*, and Car contents of line 2040 were higher than CK.

**Table 2:** The dynamic changes in chlorophyll *a* (Chl *a*), chlorophyll *b* (Chl *b*), and carotenoid (Car) contents of leaves of eight large-spike wheat lines and one multiple-spike cultivar during the developmental stages.

Line	Item (mg g^−1^)	Developmental stages			
		Jointing	Heading	Flowering	Grain filling
CK	Chl *a*	2.00 ± 0.15^ab^	2.65 ± 0.35^abc^	2.09 ± 0.11^ab^	3.03 ± 0.12^ab^
	Chl *b*	0.52 ± 0.05^a^	0.64 ± 0.09^abc^	0.55 ± 0.02^a^	0.84 ± 0.01^a^
	Car	0.42 ± 0.02^a^	0.57 ± 0.06^a^	0.39 ± 0.02^ab^	0.56 ± 0.04^a^
2005	Chl *a*	1.74 ± 0.12^ab^	2.35 ± 0.08^bc^	1.51 ± 0.08^e^	2.36 ± 0.09^c^
	Chl *b*	0.40 ± 0.03^bc^	0.54 ± 0.02^c^	0.37 ± 0.01^d^	0.58 ± 0.01^c^
	Car	0.39 ± 0.04^a^	0.54 ± 0.01^a^	0.31 ± 0.03^d^	0.46 ± 0.02^b^
2013	Chl *a*	1.61 ± 0.08^b^	2.56 ± 0.08^abc^	1.73 ± 0.07^de^	2.63 ± 0.06^bc^
	Chl *b*	0.37 ± 0.02^c^	0.60 ± 0.01^abc^	0.46 ± 0.02^bc^	0.70 ± 0.03^b^
	Car	0.38 ± 0.02^a^	0.59 ± 0.01^a^	0.33 ± 0.02^cd^	0.53 ± 0.02^ab^
2026	Chl *a*	1.91 ± 0.22^ab^	2.86 ± 0.10^ab^	2.15 ± 0.11^ab^	2.69 ± 0.15^abc^
	Chl *b*	0.42 ± 0.05^abc^	0.68 ± 0.02^a^	0.55 ± 0.03^ab^	0.74 ± 0.04^ab^
	Car	0.44 ± 0.04^a^	0.60 ± 0.02^a^	0.40 ± 0.02^ab^	0.53 ± 0.03^ab^
2036	Chl *a*	2.03 ± 0.09^a^	2.73 ± 0.04^abc^	1.68 ± 0.21^de^	2.89 ± 0.10^ab^
	Chl *b*	0.48 ± 0.02^abc^	0.64 ± 0.01^abc^	0.40 ± 0.06^cd^	0.79 ± 0.04^ab^
	Car	0.44 ± 0.02^a^	0.57 ± 0.01^a^	0.31 ± 0.03^d^	0.52 ± 0.02^ab^
2037	Chl *a*	2.04 ± 0.09^a^	2.91 ± 0.03^a^	1.96 ± 0.09^bcd^	2.68 ± 0.15^abc^
	Chl *b*	0.50 ± 0.02^ab^	0.70 ± 0.01^a^	0.52 ± 0.02^abc^	0.76 ± 0.05^ab^
	Car	0.44 ± 0.02^a^	0.59 ± 0.00^a^	0.35 ± 0.01^bcd^	0.48 ± 0.01^ab^
2038	Chl *a*	1.89 ± 0.10^ab^	2.25 ± 0.15^c^	2.03 ± 0.12^abc^	2.74 ± 0.05^abc^
	Chl *b*	0.46 ± 0.02^abc^	0.57 ± 0.03^bc^	0.53 ± 0.02^abc^	0.75 ± 0.03^ab^
	Car	0.39 ± 0.02^a^	0.45 ± 0.03^b^	0.37 ± 0.02^abc^	0.49 ± 0.03^ab^
2039	Chl *a*	2.01 ± 0.02^ab^	2.73 ± 0.17^abc^	1.97 ± 0.04^abcd^	2.68 ± 0.15^abc^
	Chl *b*	0.47 ± 0.00^abc^	0.67 ± 0.05^abc^	0.50 ± 0.03^abc^	0.74 ± 0.03^ab^
	Car	0.43 ± 0.01^a^	0.56 ± 0.03^a^	0.37 ± 0.01^abc^	0.49 ± 0.03^ab^
2040	Chl *a*	2.12 ± 0.06^a^	2.72 ± 0.04^abc^	2.29 ± 0.05^a^	3.09 ± 0.20^a^
	Chl *b*	0.50 ± 0.01^ab^	0.67 ± 0.02^abc^	0.58 ± 0.01^a^	0.84 ± 0.06^a^
	Car	0.44 ± 0.01^a^	0.57 ± 0.01^a^	0.42 ± 0.02^a^	0.56 ± 0.03^a^

**Notes.**

Values are means ± SE (*n* = 3); means followed by different small letters in the same column are significantly different at *P* < 0.05 according to LSD test.

### DW of single spikes and other parts

**Figure 7:**
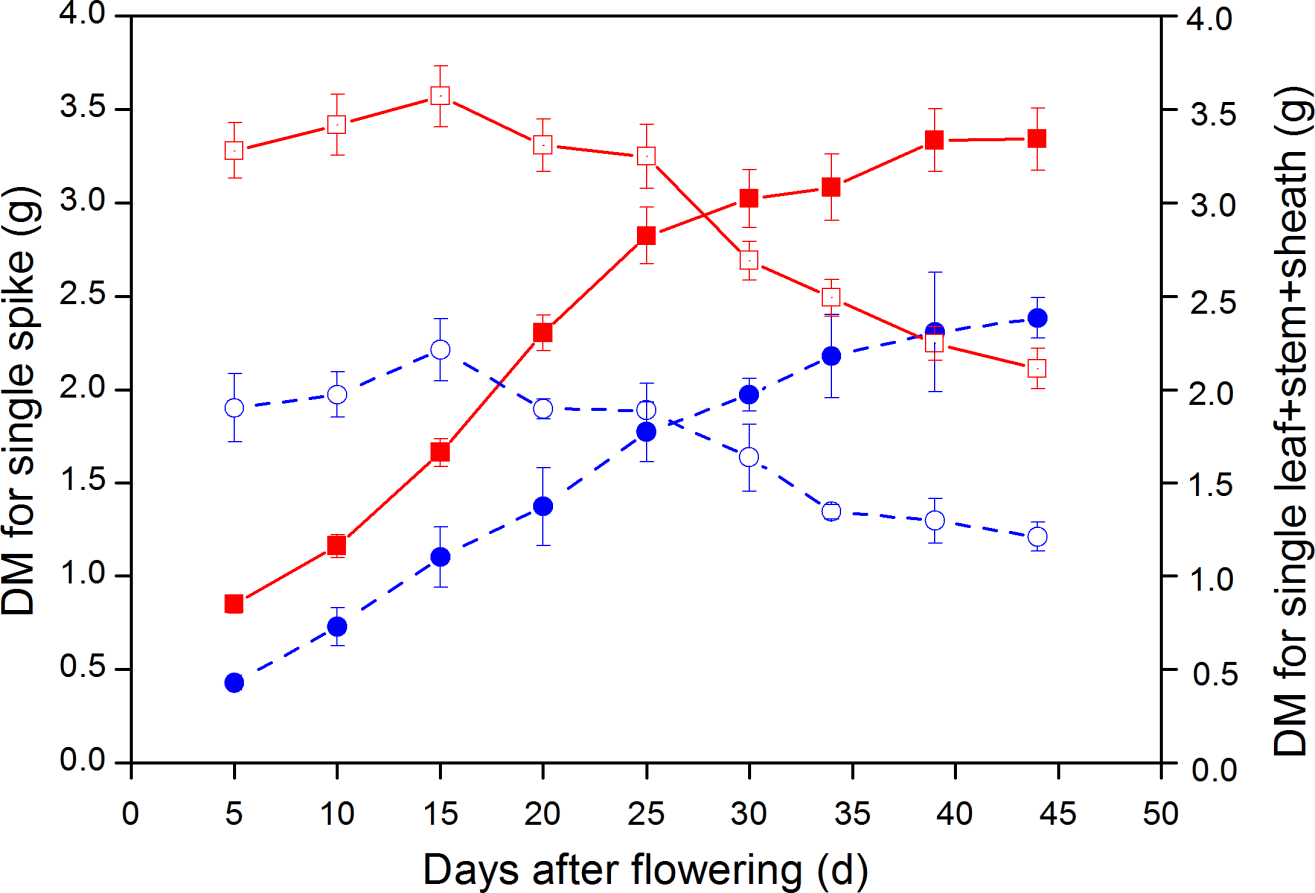
Dynamic changes in dry weight (DW) of single spike and leaf + stem + sheath in large-spike lines and CK (Xi’nong 979). ■ and • indicate DW for single spikes in large-spike lines and CK, respectively; □ and $\bigcirc$ indicate DW for leaf + stem + sheath in large-spike lines and CK, respectively.

The DW of leaf + stem + sheath for large-spike lines and CK all increased after the flowering stage; with average maximum DM reached at 15 d after flowering being 3.57 and 2.21 g plant^−1^, respectively, and then gradually declining. The DW of leaf + stem + sheath for large-spike lines and CK all reached their minimum values at 44 d after flowering: 2.11 and 1.21 g plant^−1^, respectively. The DW of leaf + stem + sheath of large-spike lines and CK began to decrease at 20 d after flowering and the photosynthetic assimilation substances were gradually transported to grain, and were reduced to 59.17 and 54.78% of the maximum DW by 44 d after flowering, respectively (Fig. 7).

The DW of wheat grain showed an “S” growth pattern from flowering to maturity; the average DMW of single spikes for large-spike lines and CK all reached maximum values 44 d after flowering: 3.34 and 2.38 g plant^−1^, respectively. The average aboveground biomass of large-spike lines at 5 d after flowering and maturity stage were 4.13 and 5.45 g plant^−1^, respectively, which were 77.03 and 51.62% higher than for CK (Fig. 7).

### Yield and primary components

The average spike length, number of grains per spike, kernel weight per spike, and 1,000-grain weight of the eight large-spike lines were significantly higher than those of CK (Xi’nong 979) (*P* < 0.05), and the number of spikes per hectare was significantly lower for large-spike lines than CK (Table 3). The yields of lines 2005, 2026, 2037, 2038, 2039, and 2040 were 3.00, 8.97, 8.33, 5.18, 5.24, and 7.03% higher than those of CK, respectively. These results indicated future positive effects for improving grain yield of large-spike lines by coordinating the relationship among agronomic traits.

**Table 3:** Differences in spike length, number of grains per spike, kernel weight per spike, number of spikes, 1,000-grain weight, and yield of eight large-spike genotypes and CK.

Cultivar	Yield traits					
	Spike length (cm)	Number of grains per spike	Kernel weight per spike (g)	Number of spikes (×10^4^ ha^−1^)	1,000-grain weight (g)	Yield (kg ha^−1^)
CK	8.2 ± 0.1^d^	43.6 ± 1.5^f^	1.9 ± 0.1^d^	445.0 ± 19.6^a^	42.4 ± 0.4^g^	6,463.4 ± 258.0^a^
2,005	13.8 ± 0.2^a^	75.1 ± 4.2^a^	3.7 ± 0.2^a^	269.2 ± 7.5^b^	49.7 ± 1.3^e^	6,657.4 ± 235.9^a^
2,013	10.5 ± 0.1^c^	57.0 ± 2.1^de^	3.1 ± 0.1^b^	266.7 ± 11.0^b^	55.8 ± 0.4^a^	6,324.3 ± 381.1^a^
2,026	14.2 ± 0.2^a^	58.2 ± 2.5^cde^	3.0 ± 0.1^b^	294.2 ± 9.4^b^	52.4 ± 0.4^bc^	7,042.9 ± 134.4^a^
2,036	10.2 ± 0.1^c^	51.3 ± 2.1^e^	2.6 ± 0.1^c^	291.7 ± 10.5^b^	51.3 ± 0.3^cd^	6,117.8 ± 211.9^a^
2,037	14.1 ± 0.2^a^	65.0 ± 2.8^bc^	3.2 ± 0.1^b^	280.0 ± 8.1^b^	50.1 ± 0.4^de^	7,001.8 ± 282.3^a^
2,038	10.6 ± 0.1^c^	59.1 ± 2.4^bcd^	3.1 ± 0.1^b^	281.7 ± 7.0^b^	52.3 ± 0.3^bc^	6,798.2 ± 285.8^a^
2,039	12.2 ± 0.1^b^	65.9 ± 2.0^b^	2.8 ± 0.1^bc^	301.7 ± 23.6^b^	44.3 ± 0.2^f^	6,801.7 ± 493.6^a^
2,040	13.7 ± 0.2^a^	54.8 ± 2.0^de^	2.9 ± 0.1^bc^	284.2 ± 11.7^b^	53.2 ± 0.2^b^	6917.6 ± 240.5^a^

**Notes.**

Values are means ± SE (*n* = 3); means followed by different small letters in the same column are significantly different at *P* < 0.05 according to LSD test.

## DISCUSSION

The carbon assimilation capacity and dry matter accumulation level of wheat plants at the jointing stage have an important influence on the number of spikes, growth, and development at later periods. This study indicated different tillering peaks for different wheat lines, and the materials had one or two tillering peaks. At the mature stage, CK had twice the number of tillers compared with large-spike lines, showing obvious differences in tiller death rate between the large-spike lines and CK. The reason for this may be the large individual characteristics and storage capacity of large-spike wheat, in which most of the photosynthetic products made by the leaves might be used to supply the grain and self-growth—this would cause the low tillering rates due to nutritional deficiency, according to research on the accumulation by Wang, Chen & Shangguan (2016). Therefore, the tiller numbers of large-spike lines should be improved by regulating row spacing, density, sowing date (Guo et al., 2009; Wang et al., 2010). This will be the key to enhancing photosynthetic performance, increasing the tillering rate, and taking full advantage of spike grain weight for realizing high yield of large-spike wheat.

Loss of green (reduced chlorophyll content) in leaves was the first symptom of leaf senescence, and the duration of leaf life differed at different plant positions, as shown in SPAD differences. The leaf photosynthesis function duration at the late growth period was closely related to grain yield, and yield might be improved by the extension of leaf photosynthesis function duration (Xie, Mayes & Sparkes, 2016). This study showed two periods of SPAD changes in intact leaves—relatively steady and rapidly declining phases—at different positions of plants for different wheat materials. When leaves were detached from plants, the chlorophyll relative values continuously decreased; however, Cao et al. (2001) showed a similar change tendency for fully expanded and detached leaves in rice, and different crops may have different processes of decline. The “loss in green” process was faster for detached than fully expanded leaves, because the chlorophyll content of detached leaves may be more easily degraded, but the fully expanded leaves might be regulated by the plant body and other organs. The SPAD for each leaf position, detached leaves, and fully expanded leaves differed among the different wheat lines.

The ability to maintain green leaf area duration during grain filling is one important physiological trait with implications for yield potential related to increasing assimilate (i.e., source) availability. For the nine wheat lines in our study, the duration of SPAD values for the flag leaf were much shorter than those for leaf 5 and leaf 7 after leaves were fully expanded (leaf 7 > leaf 5 > flag leaf); however, when leaves were detached, the durations of SPAD values were in the order of leaf 5 > flag leaf > leaf 7. Yang et al. (2014) reported that the rate of decrease in SPAD readings increased with leaf age, as leaves were physiologically older in the lower than in the upper canopy, and the rate of decrease in SPAD readings was faster in lower leaves. Chl degradation is regulated by phytochrome, and continuously accelerating rate of leaves photosynthetic pigments at different position is the factor responsible for the difference in SPAD readings (Mielke, Schaffer & Schilling, 2012). This study also showed that the Chl *a*, Chl *b*, and carotenoid contents of large-spike lines were higher than CK at heading, flowering, and grain-filling stages. In addition to the difference in shading degree, the rates of decrease in SPAD readings for different leaf positions may be influenced by other environmental factors such as ambient temperature and humidity.

Water conditions influence photosynthetic activity of photosystem II by affecting the steady state contents of its primary functional protein complexes (Slabbert & Kruger, 2011). Varieties with high RWC under stress conditions show high drought tolerance and yield (Belkheiri & Mulas, 2013). Our study showed that the RWC and *P*_n_ for wheat materials showed a declining trend after being detached from the plant. At the jointing stage, lines 2037, 2038, and 2040 had a lower rate of decline of leaf RWC and a longer average duration time of *P*_n_ than CK, and so showed strong drought resistance. At the flowering stage, materials had a stronger rate of decline than at jointing stage; lines 2005, 2013, and 2026 maintained a good water balance and long photosynthetic duration. At the grain-filling stage, the leaf RWC of materials remained within 78%–90%; and lines 2036, 2038, and 2040 showed strong drought resistance and good water retaining capacity. Differences in RWC may be attributed to differences in their ability to absorb more water from the soil and or control water loss through the stomata during growth, it may also be a result of their varied genetic ability to absorb water in the existing rooting zone and or extending rooting depth to increase water reserve for crops (Siddique, Hamid & Islam, 2000; Blokhina, Virolainen & Fagerstedt, 2003). The range of tolerance to dehydration would depend on the species and stage of development (Saint Pierre et al., 2012). For further progress in breeding for drought resistance, we will focus on subtle cultivar-level differences in expression of the gene networks involved in stress adaptation, and so improve final wheat yield.

Photosynthesis is among the plant physiological processes most sensitive to variations in soil moisture. Some researchers have shown that large-spike cultivars have more advantages in photosynthetic capacity and root activity than multiple-spike cultivars, and their carbohydrates storage capacity is large (Guo et al., 2009). The absorption of water and nutrients in maize roots was closely related to aboveground physiological processes, and strong root activity benefited the root system in absorbing water (Zhou et al., 2008). The degree of root activity indicated the ability of the root system to absorb water and transport water from the soil, and this influenced the leaf gas exchange parameters. Our pooled analysis showed a strong linear and positive correlation between *P*_n_ and root activity of large-spike lines at jointing, flowering, and grain-filling stages in a rainfed environment, consistent with previous studies in rice and maize (Yan et al., 2003; Zhou et al., 2008). This indicated that *P*_n_ might be a good tool for indirect assessment of root activity.

Wheat yield mainly comes from photosynthetic carbon assimilation after heading. Our study showed that the DW of leaf + stem + sheath and single spikes was significantly higher than CK they increased after the flowering stage and then declined as the photosynthetic assimilation substances were gradually transported to grain; the DW of wheat grain experienced an “S” pattern of growth from flowering to maturity. The average aboveground biomass at 5 d after flowering and maturity stages of large-spike lines were 77.03 and 51.62% higher than that of CK, respectively, similar to results of some researchers (Yang et al., 2002; Wang & Shangguan, 2015). Improving crop biomass is the material base for very high yields, and the leaf assimilation product of large-spike lines may better satisfy the demand for grain filling, thus reducing the storage material in the leaf + stem + sheath prematurely transferred to grain and so increase grain yield.

In this study, based on the characteristics of wheat materials have been described in the first part of materials and methods, the average spike length, number of grains per spike, kernel weight per spike, and 1,000-grain weight were significantly higher for the eight large-spike lines than CK, and the number of spikes per hectare of large-spike lines was significantly lower than for CK. The yields of lines 2005, 2026, 2037, 2038, 2039, and 2040 were higher than those of CK. Our results agreed with previous reports of the primary differences between large-spike and multiple-spike cultivars (Jiang et al., 2000; Sui et al., 2010; Lu et al., 2015; Wang, Chen & Shangguan, 2016). The large-spike lines maintained a higher production possibly by strengthening plant straw so that they appeared larger and had a smaller plant population than CK. These results indicated positive effects for improving future grain yield of large-spike lines by coordinating the relationship among agronomic traits.

## Conclusions

This study investigated the leaf photosynthetic function duration of different leaf positions among large-spike lines and a multiple-spike cultivar during wheat yield formation. It was concluded that the average spike length, number of grains per spike, kernel weight per spike, and 1,000-grain weight were significantly higher for the eight large-spike lines than CK. The large-spike lines had a slow rate of decline in RWC, long average duration time of *P*_n_, and higher yield than Xi’nong 979. There was a strong linear and positive correlation between photosynthetic rate and root activity at jointing, flowering, and grain-filling stages. The new large-spike lines might have advantages in individual plant development and growth, good drought-resistant capacity, and increasing grain yield in rainfed regions of China.

##  Supplemental Information

10.7717/peerj.5532/supp-1 Supplemental Information 1 Raw data of Figure 2-7 Figure 2: The dynamic changes in tiller number in eight large-spike wheat lines and one multiple-spike cultivar (Xi’nong 979) from seeding to mature stage Figure 3: Time courses of chlorophyll relative value (SPAD) decline in fully expanded (A–C) and detached (D–F) leaves at different positions (leaf 5, leaf 7, and flag leaf) for different wheat materials Figure 4: Time courses of relative water content (RWC) decline in detached leaves at jointing, flowering, and grain-filling stages for different wheat materials Figure 5: Time courses of photosynthetic rate (Pn) decline in detached leaves at jointing, flowering, and grain-filling stages for different wheat materials Figure 6: The relationship between photosynthetic rate (Pn) and root activity (TTC) of large-spike wheat lines during the growth period Figure 7: Dynamic changes in dry weight (DW) of single spike and leaf + stem + sheath in large-spike lines and CK (Xi’nong 979)

## Additional Information and Declarations

### Data Availability

The following information was supplied regarding data availability: The raw data are provided in the Supplemental File.
